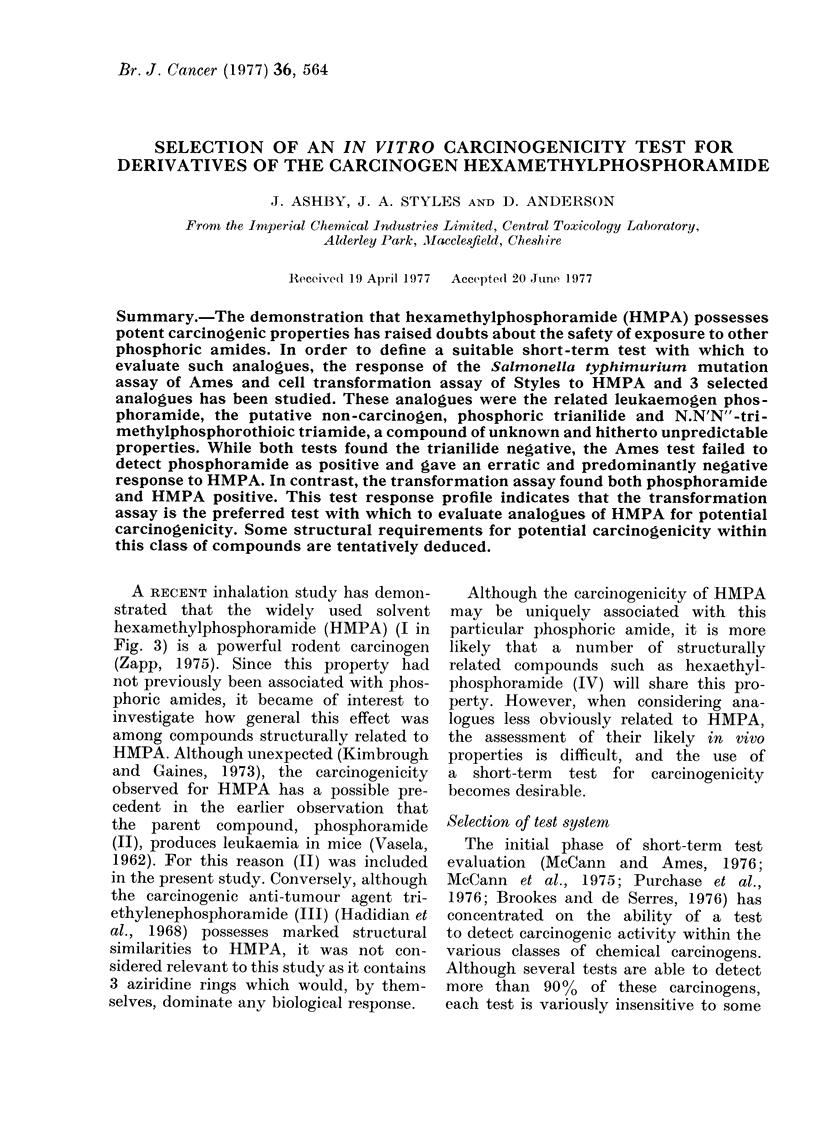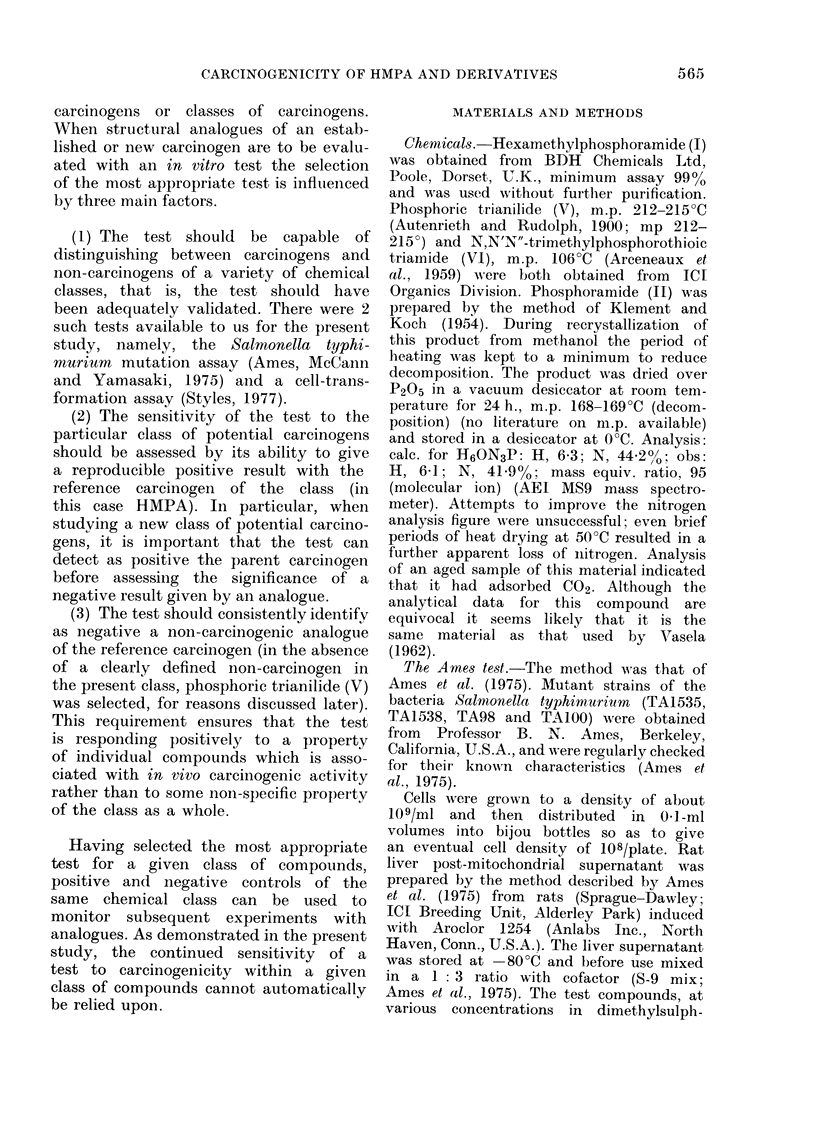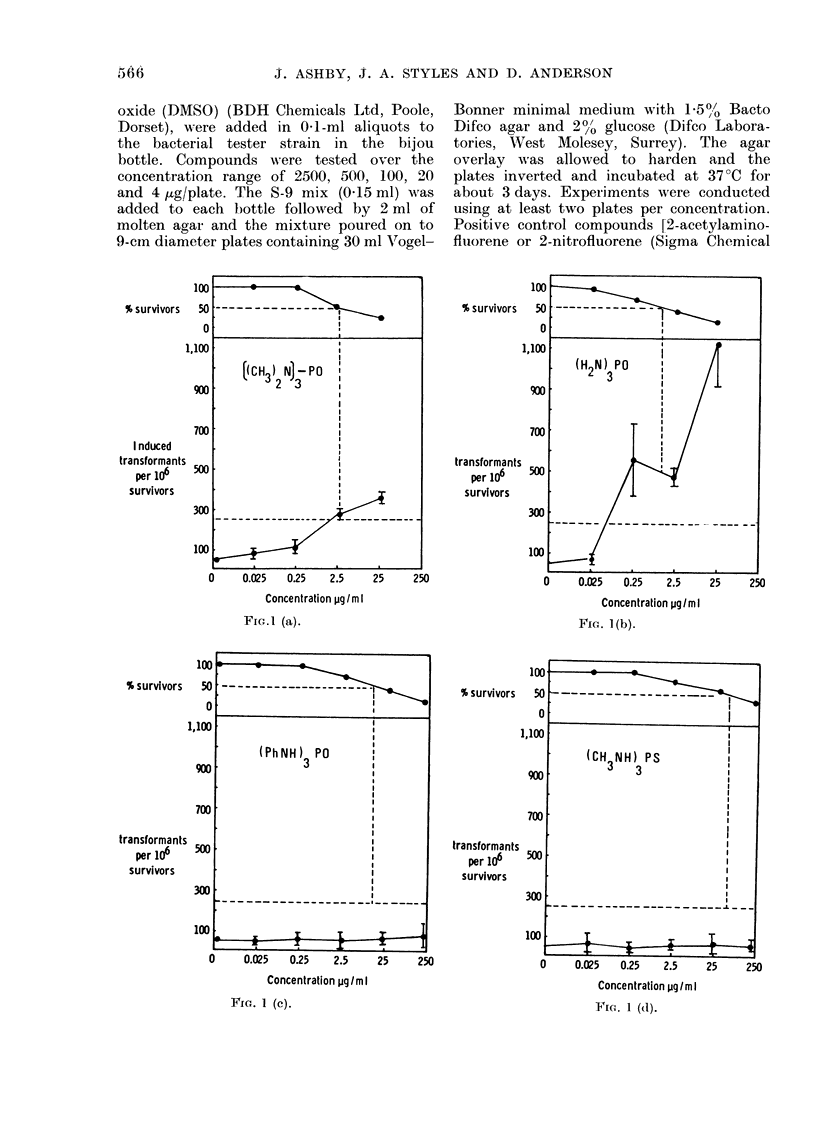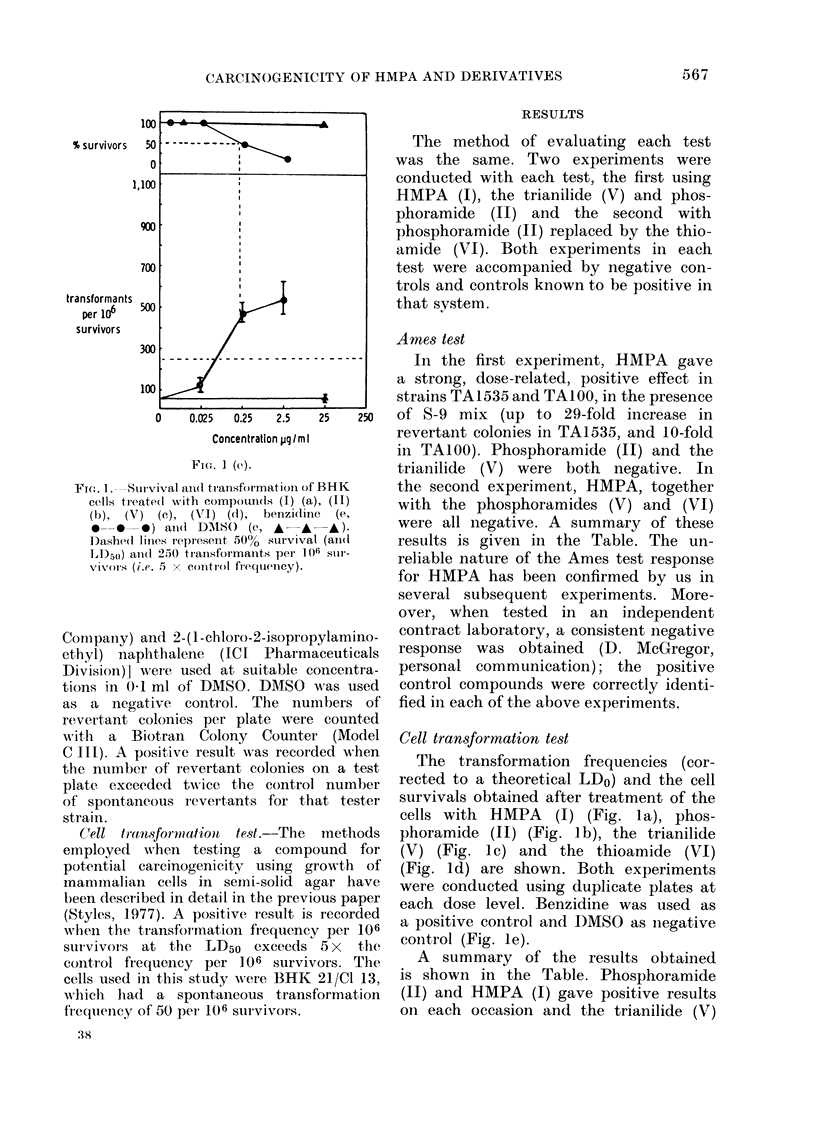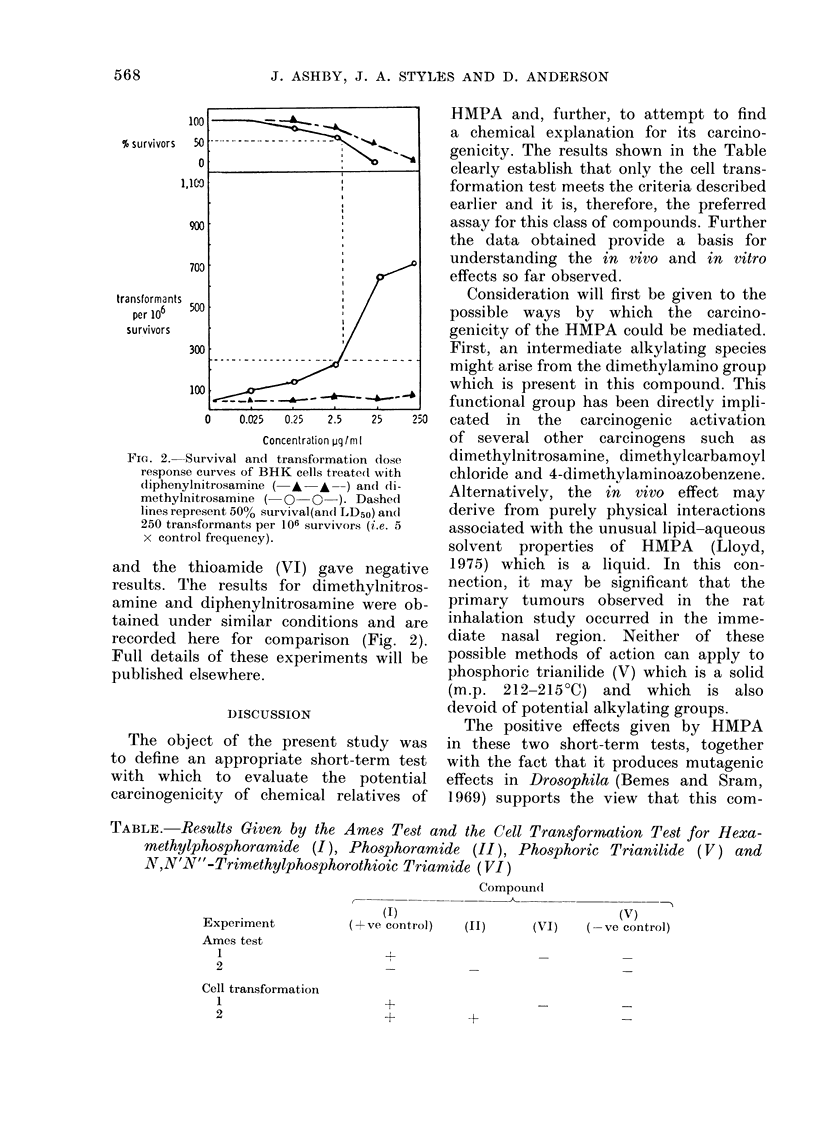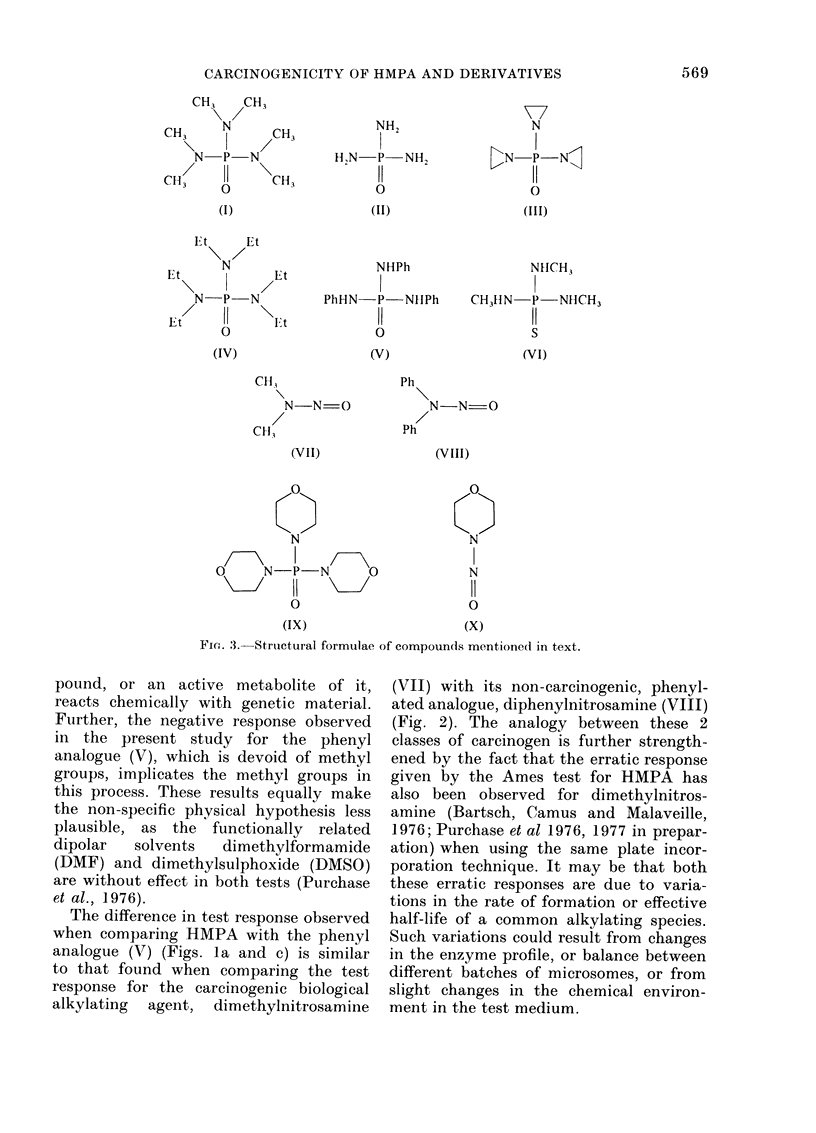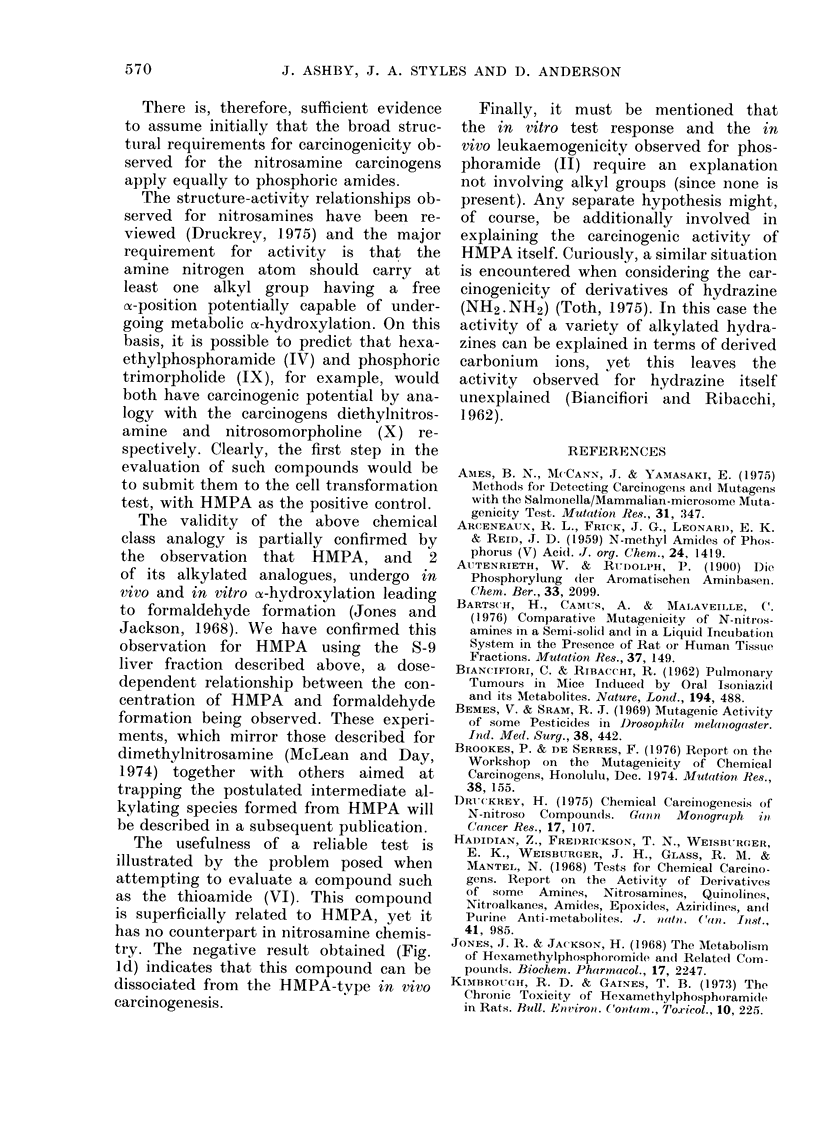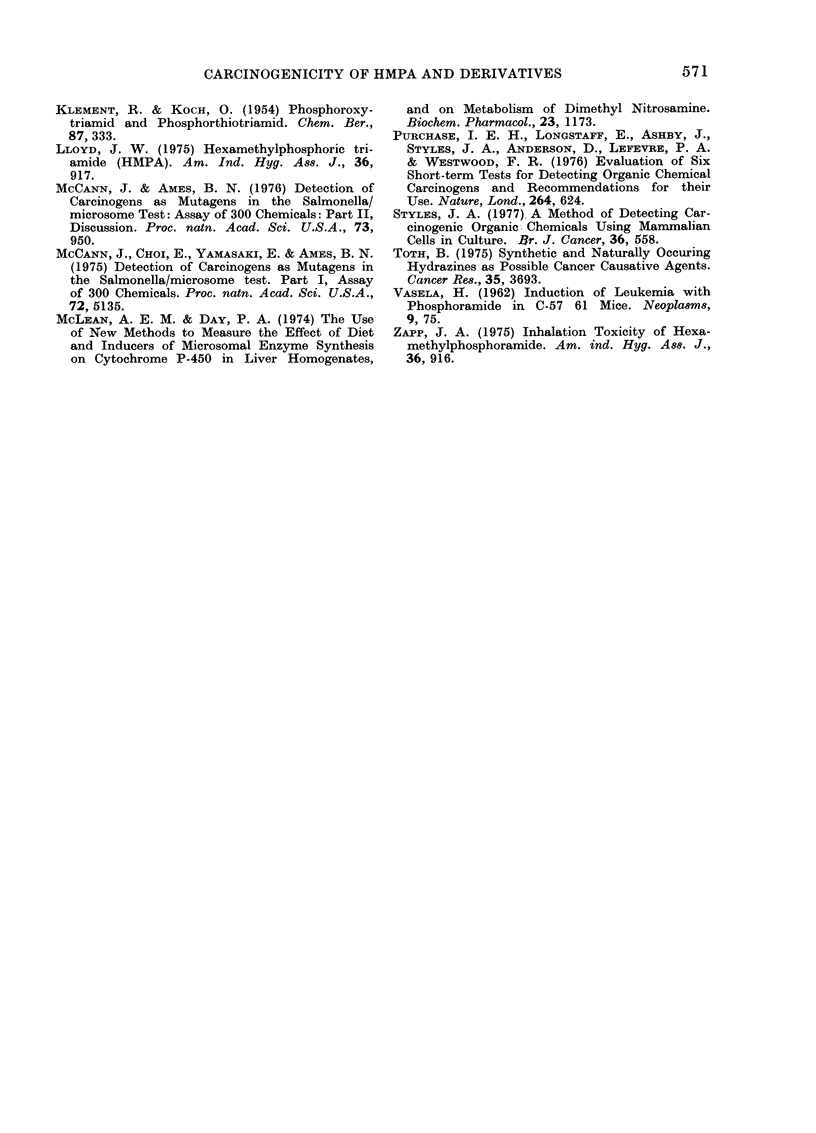# Selection of an in vitro carcinogenicity test for derivatives of the carcinogen hexamethylphosphoramide.

**DOI:** 10.1038/bjc.1977.232

**Published:** 1977-11

**Authors:** J. Ashby, J. A. Styles, D. Anderson

## Abstract

The demonstration that hexamethylphosphoramide (HMPA) possesses potent carcinogenic properties has raised doubts about the safety of exposure to other phosphoric amides. In order to define a suitable short-term test with which to evaluate such analogues, the response of the Salmonella typhimurium mutation assay of Ames and cell transformation assay of Styles to HMPA and 3 selected analogues has been studied. These analogues were the related leukaemogen phosphoramide, the putative non-carcinogen, phosphoric trianilide and N.N'N''-trimethylphosphorothioic triamide, a compound of unknown and hitherto unpredictable properties. While both tests found the trianilide negative, the Ames test failed to detect phosphoramide as positive and gave an erratic and predominantly negative response to HMPA. In contrast, the transformation assay found both phosphoramide and HMPA positive. This test response profile indicates that the transformation assay is the preferred test with which to evaluate analogues of HMPA for potential carcinogenicity. Some structural requirements for potential carcinogenicity within this class of compounds are tentatively deduced.


*Br. J. Cancer* (1977) **36**, 564

### SELECTION OF AN *IN VITRO* CARCINOGENICITY TEST FOR DERIVATIVES OF THE CARCINOGEN HEXAMETHYLPHOSPHORAMIDE

J. ASHBY, J. A. STYLES AND D. ANDERSON

*From the Imperial Chemical Industries Limited, Central Toxicology Laboratory, Alderley Park, Macclesfield, Cheshire*

Received 19 April 1977 Accepted 20 June 1977

**Summary.—The demonstration that hexamethylphosphoramide (HMPA) possesses potent carcinogenic properties has raised doubts about the safety of exposure to other phosphoric amides. In order to define a suitable short-term test with which to evaluate such analogues, the response of the *Salmonella typhimurium* mutation assay of Ames and cell transformation assay of Styles to HMPA and 3 selected analogues has been studied. These analogues were the related leukaemogen phosphoramide, the putative non-carcinogen, phosphoric trianilide and N.N'N''-trimethylphosphorothioic triamide, a compound of unknown and hitherto unpredictable properties. While both tests found the trianilide negative, the Ames test failed to detect phosphoramide as positive and gave an erratic and predominantly negative response to HMPA. In contrast, the transformation assay found both phosphoramide and HMPA positive. This test response profile indicates that the transformation assay is the preferred test with which to evaluate analogues of HMPA for potential carcinogenicity. Some structural requirements for potential carcinogenicity within this class of compounds are tentatively deduced.**

A RECENT inhalation study has demonstrated that the widely used solvent hexamethylphosphoramide (HMPA) (I in Fig. 3) is a powerful rodent carcinogen (Zapp, 1975). Since this property had not previously been associated with phosphoric amides, it became of interest to investigate how general this effect was among compounds structurally related to HMPA. Although unexpected (Kimbrough and Gaines, 1973), the carcinogenicity observed for HMPA has a possible precedent in the earlier observation that the parent compound, phosphoramide (II), produces leukaemia in mice (Vasela, 1962). For this reason (II) was included in the present study. Conversely, although the carcinogenic anti-tumour agent triethylenephosphoramide (III) (Hadidian *et al.*, 1968) possesses marked structural similarities to HMPA, it was not considered relevant to this study as it contains 3 aziridine rings which would, by themselves, dominate any biological response.

Although the carcinogenicity of HMPA may be uniquely associated with this particular phosphoric amide, it is more likely that a number of structurally related compounds such as hexaethylphosphoramide (IV) will share this property. However, when considering analogues less obviously related to HMPA, the assessment of their likely *in vivo* properties is difficult, and the use of a short-term test for carcinogenicity becomes desirable.

*Selection of test system*

The initial phase of short-term test evaluation (McCann and Ames, 1976; McCann *et al.*, 1975; Purchase *et al.*, 1976; Brookes and de Serres, 1976) has concentrated on the ability of a test to detect carcinogenic activity within the various classes of chemical carcinogens. Although several tests are able to detect more than 90% of these carcinogens, each test is variously insensitive to some carcinogens or classes of carcinogens. When structural analogues of an established or new carcinogen are to be evaluated with an *in vitro* test the selection of the most appropriate test is influenced by three main factors.

(1) The test should be capable of distinguishing between carcinogens and non-carcinogens of a variety of chemical classes, that is, the test should have been adequately validated. There were 2 such tests available to us for the present study, namely, the *Salmonella typhimurium* mutation assay (Ames, McCann and Yamasaki, 1975) and a cell-transformation assay (Styles, 1977).

(2) The sensitivity of the test to the particular class of potential carcinogens should be assessed by its ability to give a reproducible positive result with the reference carcinogen of the class (in this case HMPA). In particular, when studying a new class of potential carcinogens, it is important that the test can detect as positive the parent carcinogen before assessing the significance of a negative result given by an analogue.

(3) The test should consistently identify as negative a non-carcinogenic analogue of the reference carcinogen (in the absence of a clearly defined non-carcinogen in the present class, phosphoric trianilide (V) was selected, for reasons discussed later). This requirement ensures that the test is responding positively to a property of individual compounds which is associated with *in vivo* carcinogenic activity rather than to some non-specific property of the class as a whole.

Having selected the most appropriate test for a given class of compounds, positive and negative controls of the same chemical class can be used to monitor subsequent experiments with analogues. As demonstrated in the present study, the continued sensitivity of a test to carcinogenicity within a given class of compounds cannot automatically be relied upon.

### MATERIALS AND METHODS

*Chemicals.*—Hexamethylphosphoramide (I) was obtained from BDH Chemicals Ltd, Poole, Dorset, U.K., minimum assay 99% and was used without further purification. Phosphoric trianilide (V), m.p. 212–215°C (Autenrieth and Rudolph, 1900; mp 212–215°) and N,N′N″-trimethylphosphorothioic triamide (VI), m.p. 106°C (Arceneaux *et al.*, 1959) were both obtained from ICI Organics Division. Phosphoramide (II) was prepared by the method of Klement and Koch (1954). During recrystallization of this product from methanol the period of heating was kept to a minimum to reduce decomposition. The product was dried over $P_2O_5$ in a vacuum desiccator at room temperature for 24 h., m.p. 168–169°C (decomposition) (no literature on m.p. available) and stored in a desiccator at 0°C. Analysis: calc. for $H_6ON_3P$: H, 6·3; N, 44·2%; obs: H, 6·1; N, 41·9%; mass equiv. ratio, 95 (molecular ion) (AEI MS9 mass spectrometer). Attempts to improve the nitrogen analysis figure were unsuccessful; even brief periods of heat drying at 50°C resulted in a further apparent loss of nitrogen. Analysis of an aged sample of this material indicated that it had adsorbed $CO_2$. Although the analytical data for this compound are equivocal it seems likely that it is the same material as that used by Vasela (1962).

*The Ames test.*—The method was that of Ames *et al.* (1975). Mutant strains of the bacteria *Salmonella typhimurium* (TA1535, TA1538, TA98 and TA100) were obtained from Professor B. N. Ames, Berkeley, California, U.S.A., and were regularly checked for their known characteristics (Ames *et al.*, 1975).

Cells were grown to a density of about $10^9$/ml and then distributed in 0·1-ml volumes into bijou bottles so as to give an eventual cell density of $10^8$/plate. Rat liver post-mitochondrial supernatant was prepared by the method described by Ames *et al.* (1975) from rats (Sprague–Dawley; ICI Breeding Unit, Alderley Park) induced with Aroclor 1254 (Anlabs Inc., North Haven, Conn., U.S.A.). The liver supernatant was stored at −80°C and before use mixed in a 1 : 3 ratio with cofactor (S-9 mix; Ames *et al.*, 1975). The test compounds, at various concentrations in dimethylsulphoxide (DMSO) (BDH Chemicals Ltd, Poole, Dorset), were added in 0·1-ml aliquots to the bacterial tester strain in the bijou bottle. Compounds were tested over the concentration range of 2500, 500, 100, 20 and 4 $\mu$g/plate. The S-9 mix (0·15 ml) was added to each bottle followed by 2 ml of molten agar and the mixture poured on to 9-cm diameter plates containing 30 ml Vogel–Bonner minimal medium with 1·5% Bacto Difco agar and 2% glucose (Difco Laboratories, West Molesey, Surrey). The agar overlay was allowed to harden and the plates inverted and incubated at 37°C for about 3 days. Experiments were conducted using at least two plates per concentration. Positive control compounds [2-acetylamino-fluorene or 2-nitrofluorene (Sigma Chemical

Fig.1 (a).

Fig. 1(b).

Fig. 1 (c).

Fig. 1 (d).

FIG. 1 (e).

FIG. 1.—Survival and transformation of BHK cells treated with compounds (I) (a), (II) (b), (V) (c), (VI) (d), benzidine (e, ●—●—●) and DMSO (e, ▲—▲—▲). Dashed lines represent 50% survival (and $LD_{50}$) and 250 transformants per $10^6$ survivors (*i.e.* 5 × control frequency).

Company) and 2-(1-chloro-2-isopropylamino-ethyl) naphthalene (ICI Pharmaceuticals Division)] were used at suitable concentrations in 0·1 ml of DMSO. DMSO was used as a negative control. The numbers of revertant colonies per plate were counted with a Biotran Colony Counter (Model C III). A positive result was recorded when the number of revertant colonies on a test plate exceeded twice the control number of spontaneous revertants for that tester strain.

*Cell transformation test.*—The methods employed when testing a compound for potential carcinogenicity using growth of mammalian cells in semi-solid agar have been described in detail in the previous paper (Styles, 1977). A positive result is recorded when the transformation frequency per $10^6$ survivors at the $LD_{50}$ exceeds 5× the control frequency per $10^6$ survivors. The cells used in this study were BHK 21/Cl 13, which had a spontaneous transformation frequency of 50 per $10^6$ survivors.

### RESULTS

The method of evaluating each test was the same. Two experiments were conducted with each test, the first using HMPA (I), the trianilide (V) and phosphoramide (II) and the second with phosphoramide (II) replaced by the thioamide (VI). Both experiments in each test were accompanied by negative controls and controls known to be positive in that system.

### *Ames test*

In the first experiment, HMPA gave a strong, dose-related, positive effect in strains TA1535 and TA100, in the presence of S-9 mix (up to 29-fold increase in revertant colonies in TA1535, and 10-fold in TA100). Phosphoramide (II) and the trianilide (V) were both negative. In the second experiment, HMPA, together with the phosphoramides (V) and (VI) were all negative. A summary of these results is given in the Table. The unreliable nature of the Ames test response for HMPA has been confirmed by us in several subsequent experiments. Moreover, when tested in an independent contract laboratory, a consistent negative response was obtained (D. McGregor, personal communication); the positive control compounds were correctly identified in each of the above experiments.

### *Cell transformation test*

The transformation frequencies (corrected to a theoretical $LD_0$) and the cell survivals obtained after treatment of the cells with HMPA (I) (Fig. 1a), phosphoramide (II) (Fig. 1b), the trianilide (V) (Fig. 1c) and the thioamide (VI) (Fig. 1d) are shown. Both experiments were conducted using duplicate plates at each dose level. Benzidine was used as a positive control and DMSO as negative control (Fig. 1e).

A summary of the results obtained is shown in the Table. Phosphoramide (II) and HMPA (I) gave positive results on each occasion and the trianilide (V)

FIG. 2.—Survival and transformation dose response curves of BHK cells treated with diphenylnitrosamine (—▲—▲—) and dimethylnitrosamine (—○—○—). Dashed lines represent 50% survival(and $LD_{50}$) and 250 transformants per $10^6$ survivors (*i.e.* 5 × control frequency).

and the thioamide (VI) gave negative results. The results for dimethylnitrosamine and diphenylnitrosamine were obtained under similar conditions and are recorded here for comparison (Fig. 2). Full details of these experiments will be published elsewhere.

### DISCUSSION

The object of the present study was to define an appropriate short-term test with which to evaluate the potential carcinogenicity of chemical relatives of HMPA and, further, to attempt to find a chemical explanation for its carcinogenicity. The results shown in the Table clearly establish that only the cell transformation test meets the criteria described earlier and it is, therefore, the preferred assay for this class of compounds. Further the data obtained provide a basis for understanding the *in vivo* and *in vitro* effects so far observed.

Consideration will first be given to the possible ways by which the carcinogenicity of the HMPA could be mediated. First, an intermediate alkylating species might arise from the dimethylamino group which is present in this compound. This functional group has been directly implicated in the carcinogenic activation of several other carcinogens such as dimethylnitrosamine, dimethylcarbamoyl chloride and 4-dimethylaminoazobenzene. Alternatively, the *in vivo* effect may derive from purely physical interactions associated with the unusual lipid–aqueous solvent properties of HMPA (Lloyd, 1975) which is a liquid. In this connection, it may be significant that the primary tumours observed in the rat inhalation study occurred in the immediate nasal region. Neither of these possible methods of action can apply to phosphoric trianilide (V) which is a solid (m.p. 212–215°C) and which is also devoid of potential alkylating groups.

The positive effects given by HMPA in these two short-term tests, together with the fact that it produces mutagenic effects in *Drosophila* (Bemes and Sram, 1969) supports the view that this com-

TABLE.—*Results Given by the Ames Test and the Cell Transformation Test for Hexamethylphosphoramide (I), Phosphoramide (II), Phosphoric Trianilide (V) and N,N'N''-Trimethylphosphorothioic Triamide (VI)*

| | Compound | | | |
|---|---|---|---|---|
| Experiment | (I) (+ve control) | (II) | (VI) | (V) (−ve control) |
| Ames test | | | | |
| 1 | + | | − | − |
| 2 | − | − | | − |
| Cell transformation | | | | |
| 1 | + | | − | − |
| 2 | + | + | | − |

FIG. 3.—Structural formulae of compounds mentioned in text.

pound, or an active metabolite of it, reacts chemically with genetic material. Further, the negative response observed in the present study for the phenyl analogue (V), which is devoid of methyl groups, implicates the methyl groups in this process. These results equally make the non-specific physical hypothesis less plausible, as the functionally related dipolar solvents dimethylformamide (DMF) and dimethylsulphoxide (DMSO) are without effect in both tests (Purchase *et al.*, 1976).

The difference in test response observed when comparing HMPA with the phenyl analogue (V) (Figs. 1a and c) is similar to that found when comparing the test response for the carcinogenic biological alkylating agent, dimethylnitrosamine (VII) with its non-carcinogenic, phenylated analogue, diphenylnitrosamine (VIII) (Fig. 2). The analogy between these 2 classes of carcinogen is further strengthened by the fact that the erratic response given by the Ames test for HMPA has also been observed for dimethylnitrosamine (Bartsch, Camus and Malaveille, 1976; Purchase *et al* 1976, 1977 in preparation) when using the same plate incorporation technique. It may be that both these erratic responses are due to variations in the rate of formation or effective half-life of a common alkylating species. Such variations could result from changes in the enzyme profile, or balance between different batches of microsomes, or from slight changes in the chemical environment in the test medium.

There is, therefore, sufficient evidence to assume initially that the broad structural requirements for carcinogenicity observed for the nitrosamine carcinogens apply equally to phosphoric amides.

The structure-activity relationships observed for nitrosamines have been reviewed (Druckrey, 1975) and the major requirement for activity is that the amine nitrogen atom should carry at least one alkyl group having a free $\alpha$-position potentially capable of undergoing metabolic $\alpha$-hydroxylation. On this basis, it is possible to predict that hexaethylphosphoramide (IV) and phosphoric trimorpholide (IX), for example, would both have carcinogenic potential by analogy with the carcinogens diethylnitrosamine and nitrosomorpholine (X) respectively. Clearly, the first step in the evaluation of such compounds would be to submit them to the cell transformation test, with HMPA as the positive control.

The validity of the above chemical class analogy is partially confirmed by the observation that HMPA, and 2 of its alkylated analogues, undergo *in vivo* and *in vitro* $\alpha$-hydroxylation leading to formaldehyde formation (Jones and Jackson, 1968). We have confirmed this observation for HMPA using the S-9 liver fraction described above, a dose-dependent relationship between the concentration of HMPA and formaldehyde formation being observed. These experiments, which mirror those described for dimethylnitrosamine (McLean and Day, 1974) together with others aimed at trapping the postulated intermediate alkylating species formed from HMPA will be described in a subsequent publication.

The usefulness of a reliable test is illustrated by the problem posed when attempting to evaluate a compound such as the thioamide (VI). This compound is superficially related to HMPA, yet it has no counterpart in nitrosamine chemistry. The negative result obtained (Fig. 1d) indicates that this compound can be dissociated from the HMPA-type *in vivo* carcinogenesis.

Finally, it must be mentioned that the *in vitro* test response and the *in vivo* leukaemogenicity observed for phosphoramide (II) require an explanation not involving alkyl groups (since none is present). Any separate hypothesis might, of course, be additionally involved in explaining the carcinogenic activity of HMPA itself. Curiously, a similar situation is encountered when considering the carcinogenicity of derivatives of hydrazine ($NH_2.NH_2$) (Toth, 1975). In this case the activity of a variety of alkylated hydrazines can be explained in terms of derived carbonium ions, yet this leaves the activity observed for hydrazine itself unexplained (Biancifiori and Ribacchi, 1962).

### REFERENCES

Ames, B. N., McCann, J. & Yamasaki, E. (1975) Methods for Detecting Carcinogens and Mutagens with the Salmonella/Mammalian-microsome Mutagenicity Test. *Mutation Res.*, **31**, 347.

Arceneaux, R. L., Frick, J. G., Leonard, E. K. & Reid, J. D. (1959) N-methyl Amides of Phosphorus (V) Acid. *J. org. Chem.*, **24**, 1419.

Autenrieth, W. & Rudolph, P. (1900) Die Phosphorylung der Aromatischen Aminbasen. *Chem. Ber.*, **33**, 2099.

Bartsch, H., Camus, A. & Malaveille, C. (1976) Comparative Mutagenicity of N-nitrosamines in a Semi-solid and in a Liquid Incubation System in the Presence of Rat or Human Tissue Fractions. *Mutation Res.*, **37**, 149.

Biancifiori, C. & Ribacchi, R. (1962) Pulmonary Tumours in Mice Induced by Oral Isoniazid and its Metabolites. *Nature, Lond.*, **194**, 488.

Bemes, V. & Sram, R. J. (1969) Mutagenic Activity of some Pesticides in *Drosophila melanogaster*. *Ind. Med. Surg.*, **38**, 442.

Brookes, P. & de Serres, F. (1976) Report on the Workshop on the Mutagenicity of Chemical Carcinogens, Honolulu, Dec. 1974. *Mutation Res.*, **38**, 155.

Druckrey, H. (1975) Chemical Carcinogenesis of N-nitroso Compounds. *Gann Monograph in Cancer Res.*, **17**, 107.

Hadidian, Z., Fredrickson, T. N., Weisburger, E. K., Weisburger, J. H., Glass, R. M. & Mantel, N. (1968) Tests for Chemical Carcinogens. Report on the Activity of Derivatives of some Amines, Nitrosamines, Quinolines, Nitroalkanes, Amides, Epoxides, Aziridines, and Purine Anti-metabolites. *J. natn. Can. Inst.*, **41**, 985.

Jones, J. R. & Jackson, H. (1968) The Metabolism of Hexamethylphosphoromide and Related Compounds. *Biochem. Pharmacol.*, **17**, 2247.

Kimbrough, R. D. & Gaines, T. B. (1973) The Chronic Toxicity of Hexamethylphosphoramide in Rats. *Bull. Environ. Contam., Toxicol.*, **10**, 225.

KLEMENT, R. & KOCH, O. (1954) Phosphoroxytriamid and Phosphorthiotriamid. *Chem. Ber.*, **87**, 333.

LLOYD, J. W. (1975) Hexamethylphosphoric triamide (HMPA). *Am. Ind. Hyg. Ass. J.*, **36**, 917.

MCCANN, J. & AMES, B. N. (1976) Detection of Carcinogens as Mutagens in the Salmonella/microsome Test: Assay of 300 Chemicals: Part II, Discussion. *Proc. natn. Acad. Sci. U.S.A.*, **73**, 950.

MCCANN, J., CHOI, E., YAMASAKI, E. & AMES, B. N. (1975) Detection of Carcinogens as Mutagens in the Salmonella/microsome test. Part I, Assay of 300 Chemicals. *Proc. natn. Acad. Sci. U.S.A.*, **72**, 5135.

MCLEAN, A. E. M. & DAY, P. A. (1974) The Use of New Methods to Measure the Effect of Diet and Inducers of Microsomal Enzyme Synthesis on Cytochrome P-450 in Liver Homogenates, and on Metabolism of Dimethyl Nitrosamine. *Biochem. Pharmacol.*, **23**, 1173.

PURCHASE, I. E. H., LONGSTAFF, E., ASHBY, J., STYLES, J. A., ANDERSON, D., LEFEVRE, P. A. & WESTWOOD, F. R. (1976) Evaluation of Six Short-term Tests for Detecting Organic Chemical Carcinogens and Recommendations for their Use. *Nature, Lond.*, **264**, 624.

STYLES, J. A. (1977) A Method of Detecting Carcinogenic Organic Chemicals Using Mammalian Cells in Culture. *Br. J. Cancer*, **36**, 558.

TOTH, B. (1975) Synthetic and Naturally Occuring Hydrazines as Possible Cancer Causative Agents. *Cancer Res.*, **35**, 3693.

VASELA, H. (1962) Induction of Leukemia with Phosphoramide in C-57 61 Mice. *Neoplasms*, **9**, 75.

ZAPP, J. A. (1975) Inhalation Toxicity of Hexamethylphosphoramide. *Am. ind. Hyg. Ass. J.*, **36**, 916.